# Errors in Figure 2

**DOI:** 10.1001/jamanetworkopen.2026.41599

**Published:** 2026-09-30

In the Original Investigation titled “Neuropsychological Profile of Autopsy-Confirmed Chronic Traumatic Encephalopathy”^1^ published September 2, 2026, there were errors in the key for Figure 2. The colors for high-stage CTE and low-stage CTE were transposed. This article has been corrected.^1^
